# Recurrent fibroblast growth factor receptor3 fusion glioblastoma treated with pemigatinib: A case report and review of the literature

**DOI:** 10.1093/noajnl/vdae072

**Published:** 2024-05-14

**Authors:** Yen-Ting Liu, Yi-Hsing Chen, Chen-Han Chang, Hsiang-Kuang Tony Liang

**Affiliations:** Division of Radiation Oncology, Department of Oncology, National Taiwan University Hospital, College of Medicine, National Taiwan University, Taipei, Taiwan; Department of Biomedical Engineering, National Taiwan University, Taipei, Taiwan; Division of Radiation Oncology, Department of Oncology, National Taiwan University Hospital Yunlin Branch, Yunlin County 632, Taiwan; Graduate Institute of Clinical Medicine, College of Medicine, National Taiwan University, Taipei, Taiwan; Division of Neurosurgery, Department of Surgery, National Taiwan University Hospital, College of Medicine, National Taiwan University, Taipei, Taiwan; Department of Oncology, National Taiwan University Hospital Yunlin Branch, Yunlin, Taiwan; Division of Radiation Oncology, Department of Oncology, National Taiwan University Hospital, College of Medicine, National Taiwan University, Taipei, Taiwan; Department of Biomedical Engineering, National Taiwan University, Taipei, Taiwan; Department of Radiation Oncology, National Taiwan University Cancer Center, National Taiwan University Hospital, College of Medicine, National Taiwan University, Taipei, Taiwan

**Keywords:** FGFR3 fusion, neuro-oncology, pemigatinib, precision medicine, recurrent glioblastoma, targeted therapy

**This study presents an evaluation of pemigatinib, a selective inhibitor of FGFR1-3, in the management of recurrent FGFR3 fusion glioblastoma, underlining its potential as a precision therapy. Introduced in a 53-year-old man after standard therapies failed and genomic profiling identified an FGFR3-TACC3 fusion, pemigatinib induced a partial disease response and neurological improvement but also posed a financial burden. The case emphasizes the role of precision medicine in neuro-oncology, and supports further research into the integration of pemigatinib into glioblastoma treatment protocols.**

Glioblastoma (GB), the most aggressive and common malignant brain tumor in adults, has a poor prognosis. The 5-year relative survival rate is less than 6%. GB has been a difficult disease due to its high recurrent rate even after multimodality treatment, including gross total resection, and concurrent temozolomide chemoradiotherapy (CCRT). After local recurrence, subsequent management has little benefit. Systemic therapy-bevacizumab was granted by US Food and Drug Administration (FDA) approval for relapsed GB. It only provided improved progression-free survival. The 6-month overall survival rate was 57%.^1^

Advanced techniques like next-generation sequencing (NGS) have led to a growing focus on glioma’s molecular profiles.^2^ Distinct genetic mutations involve genes like TP53, TERT, PTEN, and fibroblast growth factor receptor (FGFR). The integration of NGS into GB research and care is guiding us towards a new era of precision medicine.

Pemigatinib, a kinase inhibitor used to treat locally advanced or metastatic, unresectable cholangiocarcinoma in adults by inhibiting FGFR1, FGFR2, and FGFR3, has been approved by the FDA. It was also used for the patients with previously treated CNS tumors in the phase II trial latterly.^3^ Herein, the study presented a patient with FGFR3 fusion GB who was treated with pemigatinib and achieved a durable disease response.

## Case Report

A 53-year-old male patient with a medical history of hypertension, type 2 diabetes mellitus, and retinal detachment presented on July 19, 2021, with an acute “spastic” sensation in the right limb and dysarthria. The magnetic resonance imaging (MRI) of the brain the following day identified a tumor in the left anterior temporal lobe and insula, approximately 4 cm in size on the axial view of the T1-weighted imaging, raising suspicions of either a brain abscess or metastasis. He then underwent a left frontotemporal craniotomy for tumor resection on July 27. The specimen was densely packed with abnormal glial cells showing rapid growth and increased cell division. It contains abnormal neurons and signs of mild damage without clear areas of dead tissue or new blood vessel growth. The glial tissue shows a high density of cells and blood vessels, with oddly shaped tumor cells and enlarged nuclei. Immunohistochemically, the dysplastic neurons are positive for S-100 protein and chromogranin A, but negative for CD34. The glial component is positive for GFAP and synaptophysin. Nuclear expression of p53 is detected in the majority of glial cells, but no IDH1 mutant is detected. The Ki-67 proliferation index is approximately 4%. The overall diagnosis was anaplastic ganglioglioma (WHO grade 3). A post-operative MRI on July 28 indicated potential residual enhancing nodules at the left inferior frontal lobe, prompting a second surgical intervention with stereotactic assistance on August 3. After surgery, he received CCRT consisting of oral temozolomide 75 mg/m2 daily along with a total radiation dose of 60 Gy in 30 fractions from August 30 to October 12, followed by adjuvant chemotherapy with temozolomide at a dose of 150 mg/m2 for the first 5 days of each month. It was completed in April 2022.

**Figure 1.**
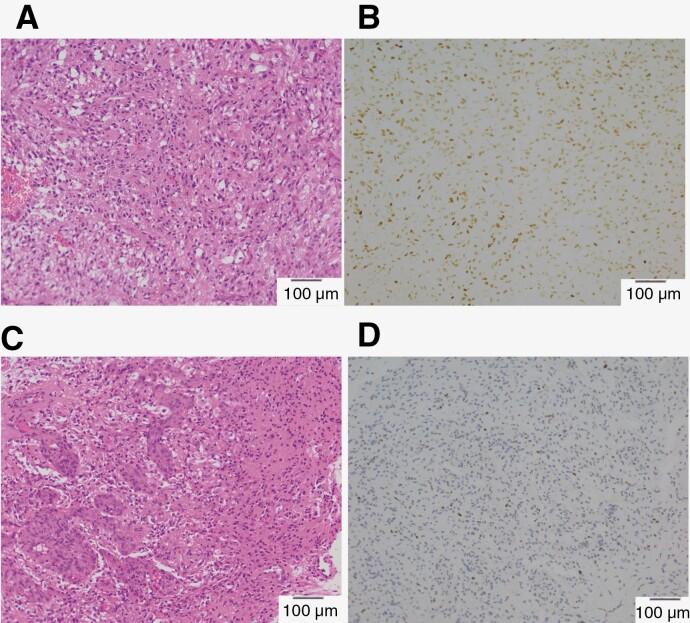
Hypercellular lesions composed of glial cells with atypia and increased mitotic activity and dysplastic neurons with the hypercellular area and adjacent parenchyma in the 200X section of hematoxylin and eosin stain (A) and immunohistochemical p53 expression in the majority of glial cells (B) at initial diagnosis. The recurrent specimens in the 200X section of hematoxylin and eosin stain showed evident microvascular proliferation and equivocal necrosis (C) and immunohistochemically, the tumors are diffusely positive for GFAP, focally positive for p53 (D).

After that, he underwent a close 3-month follow-up brain MRI. It showed stable disease until late 2022. By November of the same year, he began experiencing spasmodic episodes including muscle spasms and spasticity in the right leg. Subsequent brain MRI raised concerns of a recurrent brain tumor in the left anterior temporal lobe extending towards the insula. Concomitantly, there was a notable decline in the patient’s functional status, as measured by the Karnofsky Performance Status, which dropped from 90, indicating minimal symptoms, to 60, denoting a need for frequent assistance. This deterioration was also characterized by an intensification of headache, and a marked decrease in bilateral leg muscle strength, progressing from normal to an inability to hold against resistance within a short span of days. The 18F-Fluciclovine amino acid positron emission tomography also showed uptake in the left temporal lobe to the insula area, suggesting a recurrence. To treat this aggressive recurrence, temozolomide was restarted at a dose of 75 mg/m2 per day beginning November 30, 2022. The patient was immediately referred to the neurosurgeon. On December 27, left temporal craniotomy for tumor resection was performed, revealing GB (WHO grade 4) with positive GFAP, p53, negative IDH1, and 15%–20% of Ki-67. The pathology of the diagnosis and the recurrent specimens are presented in Figure 1. This analysis confirmed the aggressive recurrence. After a central review of the patient’s initial diagnostic specimen, IDH wild-type GB (WHO grade 4) was confirmed based on hypercellular lesions composed of glial cells with marked cytologic atypia and increased mitotic activity. In addition, immunohistochemistry showed nuclear expression of p53 in the majority of glial cells, but no IDH1 mutant was detected. During the surgery, 8 Carmustine (BCNU) 7.7 mg wafers were placed directly into the brain to target the tumor cells. Local recurrence with extensive intracranial spread of the tumor was found during surgery. The post-operative MRI of the brain on March 15, 2022, showed in-field relapse with adjacent leptomeningeal enhancement.

After reviewing the final pathology results, the patient and his wife were counseled about the poor prognosis and discussed potential second-line therapies, including re-irradiation, bevacizumab, NGS of the tumor tissue to guide targeted therapy, or optimal supportive care. With their consent, bevacizumab was started on April 13, 2023. Re-irradiation to the left temporal lobe and insular tumors was started from May 4 to May 26, 2023 with a dose of 30.6 Gy in 17 fractions.

In addition, an NGS test was performed on May 31, 2023, to screen 523 genes for variants including single nucleotide, insertion/deletion, copy number variation, fusion genes, tumor mutation burden, and microsatellite instability. The results revealed clinically actionable genomic alterations involving TERT c-124C > T, FGFR3-TACC3 fusion (FGFR3 exon 17—TACC3 exon 8), and phosphatidylinositol-4,5-bisphosphate 3-kinase catalytic subunit alpha (PI3KCA) p.V344M c.1030G > A and p.M1043T c.3128T > C (Table 1). The tumor exhibited a low tumor mutation burden and was microsatellite stable.

**Table 1.** Published Clinical Studies of FGFR Inhibitors Used for Glioma

AuthorRef	Source	Type of studies	No. of patients	Median age (range), years	FGFR alterations	Drug (dosage)	DCR	Median OS (months)
Sait, et al.^4^	MSKCC	Single center	5	8 (1.1–14)	4 FGFR1, 1 FGFR3-TACC3 fusion	Debio1347 (80 mg/1.73 m^2^ × BSA QD)	4/5 (80%)	N/A
Lassman, et al.^5^	US, Spain, Switzerl, Netherlands, Belgium	Phase 2	26	55 (20–76)	8 FGFR1, 25 FGFR3^*^	Infigratinib (125 mg days 1 to 21 of 28-day cycles)	7/26 (26.9%)	6.7 (95% CI 4.2–11.7)
Spanggaard, et al.^3^	US, Denmark, France, Germany, Israel, Italy, Japan, Korea, Spain, Switzerland, UK	Phase 2 (FIGHT-207)	13	60 (43–71)	4 FGFR1, 9 FGFR3-TACC3 fusion	Pemigatinib (13.5 mg QD)	6/13 (46.2%)	6.1 (95% CI 4.1–9.9)
NCT05267106^6^	US, Denmark, France, Germany, Italy, Japan, Netherlands, Spain, UK	Phase 2 (FIGHT-209)	83	N/A	N/A	Pemigatinib (13.5 mg QD on a 2-week on and 1-week off-therapy)	N/A (estimated completion date: November 29, 2024)	

MSKCC, Memorial Sloan Kettering Cancer Center; DCR, disease control rate; OS, overall survival; BSA, body surface area; QD, once daily; N/A, not applicable.

^*^Three patients had more than one FGFR3 alteration.

After 2 months post-irradiation, the brain MRI on July 21, 2023, showed disease progression with more leptomeningeal contrast enhancement. Additionally, the patient had little improvement in neurological symptoms. Given the suspected oncogenic driver FGFR3-TACC3 fusion leading to downstream activation of RAS and PI3K signaling, pemigatinib 13.5 mg daily on a 2-week on/1-week off schedule was recommended starting July 21, 2023. However, rectal bleeding occurred in August 2023. After consultation with the general surgeon, hemorrhoid bleeding was found by colonoscopy. Therefore, pemigatinib was continued, but bevacizumab treatment has been withheld since then. After 3 months, the follow-up MRI on October 17, 2023, showed a partial response compared to the previous MRI in July, with the patient recovering neurological function to 4/5 strength without further seizures. His Karnofsky Performance Status had returned to approximately 70 to 80.

However, with medication continued for 2 months, the patient presented with slowly progressive decreased appetite and disrupted sleep schedule. In addition to the financial burden, after discussion, they sought home hospice care with routine follow-up and stopped pemigatinib in December 2023. Overall, the patient had a good quality of life for about 6 months without disease worry after third-line treatment, almost 30 months after diagnosis.

**Figure 2.**
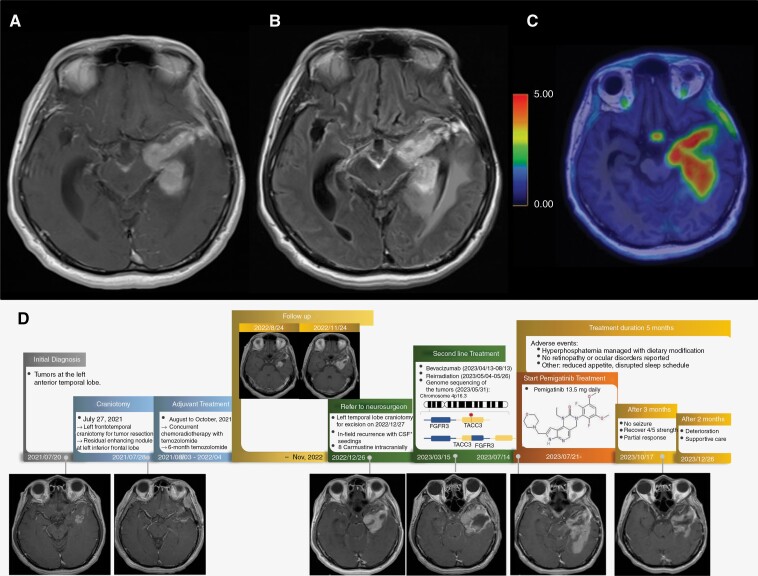
The enhancement in the left temporal lobe to the insula area with leptomeningeal seeding on the axial view of the T1-weighted with contrast (A) and T2-weighted fluid-attenuated inversion recovery with contrast (B) brain magnetic resonance imaging at recurrence before surgery. The axial view of an 18F-Fluciclovine amino acid positron emission tomography (C). Imaging series and treatment course of the patient (D).

Throughout the whole course, the toxicity of pemigatinib was mild, with hyperphosphatemia managed with nutritional counseling and dietary modification. No retinopathy or ocular disorders were reported. Only hyperphosphatemia was detected by blood testing. Ophthalmologic surveillance did not reveal retinopathy. The MRI and positron emission tomography scan during the recurrence and further details of his treatment course are summarized in Figure 2, with serial MRI images demonstrating the response.

## Discussion

Although the patient’s initial pathology showed anaplastic ganglioglioma, wild-type IDH, it is classified as WHO grade 4 based on hypercellular lesions composed of glial cells with marked cytologic atypia and increased mitotic activity without IDH1 mutant. According to the recently published study, the majority of tumors initially designated as anaplastic gangliogliomas resolved into other CNS WHO diagnoses, most commonly pleomorphic xanthoastrocytoma, GB, IDH wild-type and diffuse pediatric-type high-grade glioma by the histological review, DNA methylation profiling, and NGS.^7^ Therefore, he was disease-free only for 7 months after standard treatment including craniotomy, adjuvant CCRT, and temozolomide.

The recurrent specimen had been sent for NGS. It revealed relevant genomic alteration involving TERT (c.-124C > T), FGFR3-TACC3 fusion, and PIK3CA. Genomic landscape of GB has been investigated recently.^2^ Genetic alterations in primary tumors impact the ensuing evolution of tumor cells and the emergence of subclonal heterogeneity. The presence of TERT promoter mutations have been studied for the most common genetic alterations in GB. This mutation status is necessary and sufficient to diagnose IDH-wild-type diffuse astrocytic glioma with molecular features of GB. It is difficult to determine the exact frequency of FGFR-TACC3 fusions in GB and/or gliomas in general because of the large variation between studies. It ranges from 2% to 12%.^8^ According to the published studies for analysis of 4853 tumors by NGS, FGFR aberrations commonly affected 89% of gliomas.^9^ For infiltrative GB, 14 patients (3.6%) with FGFR mutant tumors were identified in another study.^10^ Among them, FGFR3-TACC3 fusion and FGFR3 amplification are the most common (64.3%).

The identification of the FGFR3-TACC3 fusion in various cancers, through state-of-the-art diagnostic methods such as RNA sequencing, reverse transcription polymerase chain reaction, and immunostaining, underscores its significance in the field of targeted cancer therapy. The FGFR, a receptor tyrosine kinase, is activated upon binding with its ligand, FGF, leading to the activation of critical signaling pathways such as the RAS/mitogen-activated protein kinase and PI3K pathways. TACC3 is a protein involved in mitotic spindle stabilization during cell division. Both genes are closely located on chromosome 4p16. This fusion produces a protein with a perpetually active FGFR tyrosine kinase domain, autonomously triggering the mitogen-activated protein kinase pathway without the need for ligand interaction. This misregulation results in the protein’s localization to mitotic spindle poles, causing errors in mitosis and chromosomal segregation, ultimately leading to aneuploidy, a known driver of tumorigenesis. Therefore, the FGFR3-TACC3 fusion delineates a distinct oncogenic pathway, differing from but paralleling the mechanism of ligand-dependent FGFR activation.^11,12^ Both mechanisms notably contribute to the pathophysiology of cancer through separate molecular routes.^8^ This enhanced understanding positions the FGFR3-TACC3 fusion as a critical target for the development of innovative cancer treatment strategies that specifically address the unique vulnerabilities introduced by this fusion protein, especially for GB.^3,8^

Ongoing clinical trials are delving into the effectiveness of FGFR inhibitors for tumors with specific FGFR pathway abnormalities. This research represents a critical shift towards more refined and targeted cancer therapies, aiming ultimately to improve the outcomes for patients facing solid tumors.^6^ The exploration of the FGFR3-TACC3 fusion highlights its significant potential as an effective therapeutic target. Preliminary clinical studies deploying FGFR inhibitors have yielded encouraging results, signaling a move towards more customized cancer treatment options for tumors influenced by FGFR pathway variants. Sait et al. demonstrated Debio1347, an oral FGFR inhibitor, in 5 pediatric patients with recurrent FGFR-altered gliomas, showing prolonged disease stabilization and a manageable toxicity profile.^4^ Four of the five patients had stable disease to partial response. The implications of such genetic variations, including the FGFR3-TACC3 fusion, extend beyond tumor growth and development.

Evidence from existing studies with other FGFR-driven cancers, such as urothelial carcinoma and cholangiocarcinoma, demonstrates the efficacy of pemigatinib in tumors that are refractory to conventional chemotherapeutic agents, especially FGFR3 alteration. In these cancers, pemigatinib has shown an ability to substantially inhibit tumor progression and induce partial responses, thereby extending survival in a subset of patients who have limited treatment options.^13^ The implications of these findings for GB, especially for the uncommon cases involving FGFR3 fusion genes, are intriguing.

Central to this pursuit is the identification and exploitation of molecular abnormalities that drive tumor growth. Pemigatinib’s emergence as a kinase inhibitor presents a significant development in this domain. Its mechanism, which selectively targets ATP-binding sites of FGFRs 1, 2, and 3, suggests a promising role in tackling GB subtypes that exhibit FGFR aberrations. Considering the infrequency of FGFR3 fusion events in GB, incorporating pemigatinib into existing treatment protocols requires a highly individualized, precision medicine-driven approach. However, the challenge is worth it. Yet, the blood-brain barrier poses a significant impediment to the effective delivery of many therapeutic agents into the central nervous system. Early results of pemigatinib for clinical use in intracranial tumors seem promising.^3,14^ Four of 9 (44.4%) patients with FGFR3-TACC3 fusion GB in FIGHT-207 study had control of disease, one of them had a complete response with median survival of around 6.1 months. The results are quite similar to our patients. The initial response may not last long. GB intratumoral heterogeneity and adaptive resistance mechanisms highlight an imperative need for ongoing monitoring and adaptive treatment strategies. The present study used the drug protocol of FIGHT-209,^6^ which is effective and well-tolerable for the patient. These will aim to delineate the appropriate dosages, schedules, impacts on quality of life, and potential for pemigatinib’s incorporation into multimodality treatment regimens. Published studies of the FGFR inhibitors in clinical use for glioma are listed in Table.^3–6^

Safety and efficacy are paramount for clinical use, especially in patients with GB who are often frail. FGFR inhibitors can cause side effects such as hyperphosphatemia, diarrhea, fatigue, and stomatitis, as well as ocular side effects.^15^ These side effects, particularly the risk of high blood phosphate levels, require alert monitoring and supportive care to manage any adverse effects without compromising the drug’s ability to fight cancer.

Our study was limited by one case presentation. However, FGFR alterations were centrally confirmed by NGS, and the patient followed the treatment protocol without discontinuation for 5 months. In conclusion, this study presents the first case of a patient with refractory FGFR-TACC3 fusion GB with leptomeningeal seedings who responded well to pemigatinib. This investigation of pemigatinib as a specific treatment for FGFR3 fusion GB suggests a possible shift in how we view the typically poor expectations for these patients. To fully understand the role and benefits of pemigatinib, well-designed clinical trials are essential. These efforts should provide a clear picture of how targeted therapies can effectively treat complex brain tumors such as GB, hopefully leading to better outcomes for those suffering from this serious disease.

## Supplementary Material

vdae072_suppl_Supplementary_Table
