# Correction to: Recurrent fibroblast growth factor receptor3 fusion glioblastoma treated with pemigatinib: A case report and review of the literature

**DOI:** 10.1093/noajnl/vdae116

**Published:** 2024-07-08

This is a correction to: Yen-Ting Liu, Yi-Hsing Chen, Chen-Han Chang, Hsiang-Kuang Tony Liang, Recurrent fibroblast growth factor receptor3 fusion glioblastoma treated with pemigatinib: A case report and review of the literature, Neuro-Oncology Advances, Volume 6, Issue 1, January-December 2024, https://doi.org/10.1093/noajnl/vdae072

The originally published version of this manuscript has been updated to correct the order of author Yen-Ting Liu’s affiliations.

