# Supplementary material for: Recurrent fibroblast growth factor receptor3 fusion glioblastoma treated with pemigatinib: A case report and review of the literature
Source: Neurooncol Adv. 2024 May 14;6(1):vdae072. doi: 10.1093/noajnl/vdae072 (PMC11154143; doi:10.1093/noajnl/vdae072)
Supplement: vdae072_suppl_Supplementary_Table [file vdae072_suppl_supplementary_table.docx]

Supplementary Table 1. Comprehensive genomic profile of clinically relevant genome sequencing of the patient.

| **Gene** | **Alteration** | **Transcript ID** | **Amino acid**  **change** | **Depth** | **Allele**  **frequency** | **Variant interpretation** |
| --- | --- | --- | --- | --- | --- | --- |
| ATM | SNV* | NP_000042.3 | p.(Gln95Lys) | 675 | 50.67% | Conflicting_interpretations_of_pathogenicity |
| CCND2 | SNV | NP_001750.1 | p.(Arg22Gly) | 747 | 49.40% |  |
| GABRA6 | SNV | NP_000802.2 | p.(Arg46Trp) | 669 | 52.76% |  |
| IRS2 | SNV | NP_003740.2 | p.(Gly8Glu) | 83 | 16.87% |  |
| LOC11011  7498- PIK3R3 | SNV | NP_00129035 6.1 | p.(Pro87Ser) | 632 | 44.46% |  |
| MGA | SNV | NP_00115774  5.1 | p.(Ala2594Val) | 748 | 14.31% |  |
| NSD1 | SNV | NP_071900.2 | p.(Pro2232Ser) | 957 | 56.74% |  |
| NSD1 | SNV | NP_071900.2 | p.(Arg2464Cys) | 950 | 54.74% | Uncertain_significance |
| PIK3CA | SNV | NP_006209.2 | p.(Val344Met) | 584 | 15.75% | Pathogenic |
| PIK3CA | SNV | NP_006209.2 | p.(Met1043Thr) | 696 | 17.24% | Likely_pathogenic |
| PIK3CB | SNV | NP_006210.1 | p.(Arg896Leu) | 528 | 45.64% | Likely_benign |
| RANBP2 | SNV | NP_006258.3 | p.(Gly958Cys) | 817 | 16.65% |  |
| SETD2 | SNV | NP_054878.5 | p.(Phe2125Tyr) | 747 | 18.34% |  |
| STAG2 | SNV | NP_00103621 4.1 | p.(Pro1254Arg) | 334 | 27.54% |  |
| STAT4 | SNV | NP_00123076 4.1 | p.(Leu307Phe) | 499 | 46.89% | Uncertain_significance |
| TERT | SNV | NM_198253.  2 | . | 25 | 16.00% | Likely_pathogenic |
| TP53 | SNV | NP_00111958 6.1 | p.(Gln333Glu) | 411 | 40.88% | Likely_benign |
| **Fusion Genes** | | | | | | |
| Genes | Alteration | Fusion break point | Transcript Structures | | | Supporting Reads |
| FGFR3-  TACC3 | fusion | chr4:1808661-  chr4:1737458 | FGFR3:NM_000142.4 (Exon 17)-  TACC3:NM_006342.2 (Exon 8) | | | 252 |
| FGFR3-  TACC3 | fusion | chr4:1808949-  chr4:1737347 | FGFR3:NM_000142.4 (Exon 18)-  TACC3:NM_006342.2 (Intron 7) | | | 530 |
| FGFR3-  TACC3 | fusion | chr4:1808950-  chr4:1737347 | FGFR3:NM_000142.4 (Exon 18)-  TACC3:NM_006342.2 (Intron 7) | | | 646 |
| FGFR3-  TACC3 | fusion | chr4:1808950-  chr4:1737458 | FGFR3:NM_000142.4 (Exon 18)-  TACC3:NM_006342.2 (Exon 8) | | | 479 |
| **Others** | | | | | | |
| Tumor mutational burden | | | 8.6 muts/Mb | | | |
| Microsatellite Instability | | | 1.6% Unstable Sites | | | |

*SNV, single nucleotide variant
